# The Uses of the Stomach Tube in the Practice of General Medicine1Read at the thirtieth annual meeting of the Medical Association of Central New York, held at Buffalo, October 19, 1897.

**Published:** 1898-07

**Authors:** J. A. Lichty

**Affiliations:** Clifton Springs, N. Y.


### THE USES OF THE STOMACH TUBE IN THE PRACTICE OF GENERAL MEDICINE.[1]

By J. A. LICHTY, M. Ph., M. D., Clifton Springs, N. Y.

MUCH valuable information has been gained since Kussmaul and Leube first used the stomach tube methodically in the diagnosis and treatment of diseases of the stomach. But by most men this information has been looked upon as being only for the specialist in diseases of the stomach and of little practical value

1. Read at the thirtieth annual meeting of the Medical Association of Central New York, held at Buffalo, October 19, 1897.

to one practising general medicine. The reasons for this are various :

(1) Special instruments and reagents must be employed and their proper use requires the knowledge of a certain technique. (2) Physicians in general practice feel that they cannot spare the time for " such outside work." (3) There is a prevailing opinion that the use of the stomach tube involves a procedure which, to say the least, is very disagreeable, not only to the patient but even to the doctor, and also that its use is accompanied by some degree of danger. (4) Many are of the opinion that the data obtained are of an uncertain nature and cannot be relied upon.

It is the purpose of this communication to show that these objections are, in the main, without foundation ; that the successful use of the stomach tube is within the range of one practising general medicine and that neglect of its use is depriving both physician and patient of much that is helpful.

The instruments needed are very simple : a rubber tube, 75 c.m. long and 1¼ c.m. in diameter, having two large fenestræ at the stomach end, and a glass tube, 5 c.m. long at the mouth end, to which are again attached about 6 c.m. of rubber tubing ; an ordinary Politzer's bag for aspiration and a rubber bulb with valve for inflation. With this simple outfit, a test meal can be taken, the stomach can be washed out and it can be inflated for diagnostic purposes.

It requires a little practice to pass a stomach tube easily and with the least discomfort to the patient. Before proceeding with the operation it is well to explain to the patient what you expect him to do, telling him to swallow when the tube touches the throat, to bite upon the tube when he feels as though he must vomit, to breathe deeply if he feels that he is suffocated and to bear down, as at stool, when told to do so. Biting upon the tube tends to overcome nausea and prevents the patient from ejecting the tube ; deep breathing assures the patient that he is not being suffocated and the voluntary effort of bearing down is called into service when the tube is inserted about 55 c.m. to aid in expressing the contents.

I prefer a Politzer's bag to aspirate the stomach, having been impressed with its simplicity and convenience while a student at Professor Ewald's polyclinic at the Augusta hospital, Berlin. This, with the aid of the patient's expulsive efforts, is sufficient to empty the stomach. No lubricant is needed for the stomach tube. It is distasteful to the patient and the saliva moistens the tube sufficient to cause it to slip down easily. The instruments should afterward be washed in hot water containing a small amount of lysol.

The purpose of this communication does not permit me here to pass in review and criticise in detail the different methods of chemical examination of gastric juice. Text-books on diseases of the stomach or on physical diagnosis give explicit directions on the chemical analysis.

For ordinary and practical purposes the apparatus required is no more elaborate than for the analysis of urine. The qualities to be determined and the respective reagents employed are in brief as follows:

1. Reaction is determined by means of ordinary litmus paper.

2. Free acids, Congo-red paper.

3. Free HCl, Gunsberg's phloroglucin-vanillin solution.[1] These three tests are easily made and are of prime importance.

4. The total acidity is determined by trituration with a deci-normal solution of NaHO, using phenol-phthalein as an indicator.

5. *Lactic Acid* is detected with Uffelman's reagent.[2]

6. *Pepsin* can be safely assumed to be present if free HCl is found, or a definite amount of egg-albumin can be added to a definite amount of filtered contents and digestion observed at body temperature. This test will give a fair idea of the digesting power of the gastric juice.

7. The degree of starch digestion is noted in the microscopical examination of the residue of the stomach contents, and by Lugol's test of the filtrate.

These tests, when one is once thoroughly acquainted with them, can be made very readily and will furnish an invaluable amount of information. It will not be denied that it requires some time to enter into such detail in diagnosticating disease of the stomach, but the accurate knowledge gained compensates for the time expended.

In answer to the objection to the stomach tube on account of its being disagreeable and difficult for the patient to swallow, I need only give my experience with patients. Those who need it will submit to the stomach tube without any objection or protest. Among a hundred consecutive patients during the past eighteen months, to whom I proposed using it, not one objected, and in only one case was I unable to introduce it. The cause of this failure was an existing pharyngitis which really made the operation painful. This list of a hundred patients does not include dispensary or free patients in a charity hospital, but private patients from the well-to-do and intelligent classes. With none have I noticed any alarming symptoms due to the passage of the tube during or after the operation. Within the limits prescribed by text-books there is absolutely no danger.

Having shown that the use of the stomach-tube need not be confined to the specialist alone, and that it has, therefore, a proper place among the other means used for diagnostic purposes in general medicine, the questions arise, what information is obtained from its use, what is its relative importance, and to what extent can it be relied upon in the diagnosis and treatment of diseases of the stomach?

Sidney Martin[3] says that "It is evident that the examination of the contents of the stomach during digestion is of great service in the diagnosis and treatment of disordered digestion."

Mathieu[4] says, "At the present day it is, indeed, impossible to understand the nature of or to diagnosticate dyspepsia and its clinical varieties without a practical knowledge of the new methods of examination."

Classifying, in the broadest sense, diseases of the stomach as functional and organic, what first is the information gained by the use of the stomach-tube in some of the functional diseases?

"In hyperchlorhydria, the diagnosis can be made with certainty only after repeated examination of the gastric juice."[5] It is very important to distinguish between hyperchlorhydria and other conditions of the stomach in which there is hyperacidity, for the treatment of hyperchlorhydria is entirely different from the treatment for the hyperacidity of gastric fermentation. Several times when I hoped to avoid the use of the stomach tube it revealed to me later, after unsuccessful treatment, that I was treating one for the other and vice versa.

In motor insufficiency of the stomach, methods of diagnosis without the tube have been employed, but none is so satisfactory as lavage of the stomach in the morning when the patient has fasted for twelve hours after a supper of mixed food.

In nervous dyspepsia, while writers agree that the stomach contents are normal, yet I have found here a use for the stomach tube, which to me is invaluable. It is very easy to say to a patient that his symptoms are all nervous, that strict dieting is unnecessary, but it is often a difficult matter to get him to follow out your directions long enough to accomplish any results. In treating diseases of the stomach, more than in other diseases, it is necessary to have the coöperation of the patient, and this, not only for a day, but usually for an extended time, even after the patient has passed out of your immediate care. You can secure such coöperation almost invariably by proving to your patient beyond a doubt that the digestive disturbance is of a nervous nature, and here nothing is so convincing as the examination of the stomach contents. My experience warrants me in saying that if this method were adopted by physicians generally it would not only aid in the treatment of this condition, but it would also be found that the frequency of nervous dyspepsia is not nearly as great as is usually supposed.

In organic diseases of the stomach the tube affords us important information. Unfortunately, this information has often been misconstrued, and has given rise to apparent contradictions. In 1879, Van den Velden[6] first stated that free HCl is always absent in gastric cancer. Cohn and Von Mehring[7] soon proved this to be too sweeping a statement. Ten years later, Ewald[8] declared that free HCl is absent only in the large majority of cases of gastric cancer. In 1893, Boas,[9] contrary to the usual opinion that lactic acid is present in normal digestion, asserted that it is always absent, and absent also in all affections of the stomach, except carcinoma. To him the presence or absence of free HCl in the gastric contents was of little importance in the diagnosis of gastric cancer. Also in 1895, Friedenwald,[10] in an original article on the quantitative estimation of the rennet-zymogen, attempted to show its diagnostic value in gastric cancer.

In reviewing the reported cases upon which these writers base their various opinions, I was impressed with the one prominent fact, that in a great majority of gastric cancers, free HCl is absent and that in the remaining few it is much diminished. In view of this fact, I have found myself relying very much upon the examination of the gastric contents in making a diagnosis of, or in excluding, cancer of the stomach.

In ulcer of the stomach the nature of the disease naturally forbids the use of the stomach tube.

In the diagnosis of chronic gastritis, the stomach tube is of incalculable value. Einhorn[11] in the diagnosis of this disease mentions the examination of the stomach contents as though it was as natural to resort to such a procedure as to examine the urine in suspected disease of the kidneys.

The stomach tube is necessary to determine the exact size and position of the stomach. The method of distention with bicarbonate of soda and tartaric acid is not satisfactory, since the amount of gas relative to the size of the abnormal stomach cannot be regulated and it is also sometimes a dangerous procedure. The succussion splash, described by Bouchard, and recently so elaborately applied by Dr. Boardman Reed,[12] is not always to be relied upon, while the inflation of the stomach through the tube with a rubber bulb is easily accomplished. I practice the succussion splash in all of my cases, but several times the stomach-tube has shown me that I had been led astray by the splash of a distended transverse colon.

Comparatively little is yet known of intestinal indigestion, evidently on account of the difficulty of examining the process of digestion in the bowels. Herter and Smith,[13] in their exhaustive work upon the relation of the preformed sulphates and indican to intestinal indigestion, have opened up a large field for investigation, but their methods are as yet too complicated to be made practical for clinical purposes. I have found that a knowledge of the exact condition of gastric digestion frequently aids me in the diagnosis of intestinal indigestion. If there is gastric hyperacidity, and the excessively acid chyme emptying into the bowel neutralises the alkalinity necessary for proper starch digestion, an intestinal indigestion will arise.

The uses of the stomach-tube in toxic conditions need not be mentioned here. For lavage it must sometimes be resorted to and in certain conditions, such as pyloric obstruction with dilatation, will give relief when all other means fail. It has been objected to because patients are prone to get into the habit of washing out their stomachs upon the least discomfort. Judicious care on the part of the physician will save the patient from such a habit.

In a recent lecture upon the relation of the diseases of the stomach to other diseases, Prof. Ewald denied that he was a specialist on diseases of the stomach,[14] but at the same time he declared that the definite knowledge of the diseases of the stomach now at the physician's command, makes it obligatory upon him to avail himself of this knowledge to employ the methods for obtaining it and to use it for the good of his patients. It is in behalf of such an opinion that I have tried to indicate the practical use of the stomach tube in general medicine.

### ADDENDA.

1. For a careful comparison of the different methods for estimating free HCl in gastric contents see an exhaustive article by Dr. David Linn Edsall, Univ. Med. Mag., September, 1897.

2. Dr. John P. Arnold has published a new and simple test for lactic acid in gastric contents and a quick method of estimating approximately the quantity present. Jour. Am. Med. Ass., August 21, 1897.

3. Sydney Martin, Diseases of the Stomach, p. 121.

4. Mathieu, Diseases of the Stomach and Intestines, p. 1.

5. Einhorn, Diseases of the Stomach, p. 294.

6. Deutsches Arch. f. Klin. Med., Bd. 23, p. 369.
7. Berlin Klinische Wochenschrift.
8. Ewald, Krankheiten des Magens, p. 355.
9. Münchener Med. Wochenschrift, October 24, 1893.
10. Med. News, June 22, 1895.
11. Einhorn, Diseases of the Stomach, p. 170.
12. Med. News, January 18, 1896.
13. New York Med. Jour., June and July, 1895.
14. Ewald, Unpublished Lecture.

### DISCUSSION.

Dr. BENEDICT, of Buffalo: I want to hurriedly endorse one thing the doctor has said with a proviso. He has said that the stomach tube should not be limited to the use of the specialist. If the doctor uses the word specialist in the business, commercial sense, that is perfectly correct; but if he means to imply that every man who once a year or once in two years is going to try to pass the stomach tube and examine the stomach contents, or ought to do it, I think that is a mistake. I recall two cases in which I was strongly tempted to use the stomach tube for diagnostic purposes. In one of them the patient died suddenly from hemorrhage at the very time appointed for examination. It was simply the matter of an urgency call on the part of the physician with whom I was to see the case in consultation that we did not pass the tube at that time and have a sudden death on our hands. And another just such case, except there was not the theatrical interruption. It seems to me, without reference to the specialism in a business sense, if a man is prepared to do thorough work with the use of the tube and do a good deal of it, let him by all means use the tube whether he calls himself a specialist or not. If he is simply going to use the tube once or twice a year, he better not use it at all, because it is a risky thing to use in aneurism and in cases of pregnancy where there is a chance of abortion, and in any cases of that sort where we have got to avoid a chance for spasm. Again, a man who uses a tube must make up his mind to two or three things at the start. In the first place, he cannot let that tube take the place of ordinary methods of examination. In the second place, he must not use that tube to fill the stomach up as full as it can be. Do not understand me as criticising the speaker, but simply to guard against an idea that we all possibly obtained from the paper. I think the speaker will agree with me that the man who fills the stomach just as full as he can is committing malpractice. It is not simply that we are going to kill the patient by filling up the stomach with water, but that we are going to set up a dilatation or that it is going to increase a dilatation already present. There are a good many men who cannot tell where the stomach is, or how large it is, and yet they will talk about that stomach being dilated. If a man knows where the stomach is, let him try it; if he does not know where it is, he ought not use the word dilatation, because it is simply a term. I want to say, Mr. President, that if I had on the one hand a chemical examination of the stomach contents and on the other hand a careful examination of the urine and of the blood and a thorough physical examination, including auscultory percussion, to lay out the stomach in its size and area, I would rather take the second alternative. You can get a great deal more information from those two examinations than you can from the use of the tube. A great many patients get to using the tube themselves and they do enormous mischief with it. I remember one case particularly, dilatation, a case going along nicely, but the man insisted every time he had a little fermentation of the stomach on using the tube. He lost flesh; lost nourishment; he was removing food from the stomach that he ought not to. If you will give the patient at the height of digestion, that is, an hour after a little meal, two hours after a heavy meal, hour and a half after a heavy meal, a small quantity of concentrated solution of soda and listen over the stomach, you can judge of the acidity by the sound verberations that you get, just as you can judge of the acidity if you pour a weak solution of acid into the palm of your hand with a little soda. That is an excellent test, but mind, you have got to practise it long enough so that you can distinguish the rustling of garments and the movements of the stomach; simply the churning movements. Those you must eliminate. Now if, on the other hand, we know that a patient has either hydrochloric acid or fermentation acid, in cases it is a perfectly easy matter to make the diagnosis. We can almost make them with the statement of the patient.

Dr. LICHTY: I was sorry I was not able to read the paper through. I think some of the objections which Dr. Benedict has brought out have been answered in the text. I think he does not give the general practitioner credit for enough knowledge of the disease of the stomach. I was speaking to the general practitioner and I think the general practitioner knows where the stomach is and will be able to diagnosticate or distinguish between a dilated stomach and a normal stomach. As to the use of the stomach tube—or to the bad use of the stomach tube by the patients—in my paper I have stated that the tube should not be allowed to the patient except under very limited conditions, and I think that Dr. Benedict would have changed his remarks if I could have been allowed to finish the paper. In reference to the specialist of the stomach, I might say that not many years ago, when I heard Prof. Ewald in one of his lectures at his clinic, he denied that he was a specialist in disease of the stomach and said that he was a general practitioner, but that he thought the stomach tube revealed such an amount of material to the practitioner in general that every man who practises medicine should have some knowledge of it, and as the general practitioner receives the bulk of the patients and probably one-third of them have some trouble with the stomach, he ought to avail himself of the information which can be obtained by the use of the stomach tube.

Dr. BENEDICT, of Buffalo: May I be allowed just one word? Mr. President, I think the doctor and I have each been a little hurried and have, perhaps, put things in a way that suggest some antagonism where there was no such intention. My remarks about specialists have simply to do with this point: Has a man time in which he can make a thorough study of the subject; not whether he is well informed, but can he do the good, hard grinding and put the time on the work that alone will make it valuable? That is the only point I wanted to make in that regard.

---

THE danger of bringing yellow fever into the United States through the medium of vessels used during the transportation of troops to Cuba and their return, is by no means a small one; but the navy department has determined to adopt every precautionary measure for the prevention of such accident and will thus coöperate with the marine hospital service.—*Ohio Medical Journal.*

